# Determinants of heart rate in Svalbard reindeer reveal mechanisms of seasonal energy management

**DOI:** 10.1098/rstb.2020.0215

**Published:** 2021-08-16

**Authors:** L. Monica Trondrud, Gabriel Pigeon, Steve Albon, Walter Arnold, Alina L. Evans, R. Justin Irvine, Elżbieta Król, Erik Ropstad, Audun Stien, Vebjørn Veiberg, John R. Speakman, Leif Egil Loe

**Affiliations:** ^1^ Faculty of Environmental Sciences and Natural Resource Management, Norwegian University of Life Sciences, NO-1432 Ås, Norway; ^2^ Département de Biologie, Faculté des Sciences, Université de Sherbrooke, 2500 boul. de l'Université, Sherbrooke, Québec, Canada, J1 K 2R1; ^3^ The James Hutton Institute, Craigiebuckler, Aberdeen AB15 8QH, UK; ^4^ Department of Interdisciplinary Life Sciences, Research Institute of Wildlife Ecology, University of Veterinary Medicine, Savoyenstr. 1, 1160 Vienna, Austria; ^5^ Department of Forestry and Wildlife Management, Inland Norway University of Applied Sciences, Campus Evenstad, 2418 Elverum, Norway; ^6^ Frankfurt Zoological Society, PO Box 100003, South Africa Street, Addis Ababa, Ethiopia; ^7^ School of Biological Sciences, Institute of Biological and Environmental Sciences, University of Aberdeen, Aberdeen AB24 2TZ, UK; ^8^ Faculty of Veterinary Science, Norwegian University of Life Sciences, PO Box 8146, NO-0033 Oslo, Norway; ^9^ Department of Arctic and Marine Biology, The Arctic University of Norway, PO Box 6050 Langnes, NO-9037 Tromsø, Norway; ^10^ Norwegian Institute for Nature Research, PO Box 5685 Torgarden, NO-7485 Trondheim, Norway; ^11^ Center for Energy Metabolism and Reproduction, Shenzhen Institutes of Advanced Technology, Chinese Academy of Sciences, Shenzhen 518055, People's Republic of China; ^12^ CAS Center of Excellence in Animal Evolution and Genetics, Kunming 650223, People's Republic of China; ^13^ State Key Laboratory of Molecular Developmental Biology, Institute of Genetics and Developmental Biology, Chinese Academy of Sciences, Beijing 100101, People's Republic of China

**Keywords:** activity, Arctic, plasticity, reproduction, thermoregulation, ungulates

## Abstract

Seasonal energetic challenges may constrain an animal's ability to respond to changing individual and environmental conditions. Here, we investigated variation in heart rate, a well-established proxy for metabolic rate, in Svalbard reindeer (*Rangifer tarandus platyrhynchus*), a species with strong seasonal changes in foraging and metabolic activity. In 19 adult females, we recorded heart rate, subcutaneous temperature and activity using biologgers. Mean heart rate more than doubled from winter to summer. Typical drivers of energy expenditure, such as reproduction and activity, explained a relatively limited amount of variation (2–6% in winter and 16–24% in summer) compared to seasonality, which explained 75% of annual variation in heart rate. The relationship between heart rate and subcutaneous temperature depended on individual state via body mass, age and reproductive status, and the results suggested that peripheral heterothermy is an important pathway of energy management in both winter and summer. While the seasonal plasticity in energetics makes Svalbard reindeer well-adapted to their highly seasonal environment, intraseasonal constraints on modulation of their heart rate may limit their ability to respond to severe environmental change. This study emphasizes the importance of encompassing individual state and seasonal context when studying energetics in free-living animals.

This article is part of the theme issue ‘Measuring physiology in free-living animals (Part II)’.

## 1. Introduction

The interplay between energy requirements and availability is fundamental to all living organisms [1]. Because both energy requirements and availability depend on environmental conditions, the balance between them contributes to shaping ecological interactions at the level of individuals [2], populations [3], species [4,5] and whole ecosystems [6]. Seasonal changes in energy supply have led to the evolution of life-history strategies and a wide range of physiological, morphological and behavioural adaptations that enable animals to adjust their metabolic phenotype to the prevailing season of the year [7]. While seasonal plasticity is relatively well studied, little is known about the capacity of seasonally adapted animals to respond to short-term environmental fluctuations within the season [8–10]. It has been suggested that strong phenotypic plasticity may limit the potential for evolutionary responses to climate change [11]. Thus, understanding the relationship between interseasonal and intraseasonal plasticity of metabolic adaptations is of fundamental importance for predicting animal resilience to climate change [12].

For mammals and birds, the cost of maintaining high core body temperature increases as ambient temperatures decline [13]. However, in many seasonal environments, the availability and quality of food plants decline simultaneously with decreasing ambient temperature, particularly in temperate and arctic environments [14]. Hence when herbivorous endotherms have potentially the greatest thermoregulatory demands due to low ambient temperatures, they also have the lowest supply of food to meet such demands [15]. By contrast, when food is more plentiful, animals must both reproduce and replenish energy reserves. In the most extreme seasonal environments, this period of high energy supply can be as short as two months [7]. Trade-offs between energy investment in offspring and energy acquisition to replenish reserves are often shaped by physiological [16,17] or nutritional [18] constraints.

The most pronounced temporal reductions in energy expenditure are observed in species exhibiting daily torpor and hibernation, characterized by substantially lowered metabolic rate, body temperature and reduced movement [19]. However, accumulating evidence shows that many non-hibernating temperate animals also display seasonal adjustments in metabolic rate [20–23] through reduced body temperature and activity levels [24–26]. Similar responses have been observed in desert ungulates during the hot, dry season when food is limited [27–29]. Adjustments in behavioural and physiological traits are clearly important to reduce the energetic costs in periods of low resource availability, and simultaneously maximize replenishment of body reserves and reproduction in periods of high resource availability [30].

Heart rate is a key physiological parameter of animal performance because it correlates with oxygen consumption and hence is often used as a proxy for metabolic rate [31,32]. Also, heart rate is a key biologging parameter because the heart beat generates an electrical signal readily monitored by small implantable devices, providing information on energetics of wild animals in their natural habitat over long time periods [4], at fine temporal scales [33], and in remote or harsh environments ([34,35], reviewed in [36]). Furthermore, heart rate can be influenced by locomotor activity [37,38], ambient temperatures [5,39] and reproduction [40,41]. Although substantial knowledge about the relationship between energy expenditure (indexed by heart rate) and specific physiological or environmental processes exists, few studies have explored how these potentially interact to influence variation within periods of energy surplus and deficit [5,41,42].

We quantified the effects of environmental, behavioural and physiological factors on variation in heart rate of Svalbard reindeer (*Rangifer tarandus platyrhynchus*)—a keystone species in the High Arctic with strong seasonal changes in nutrition and metabolic activity. Inhabiting a predator-free environment, they forage for up to 70% of their time in summer to meet the energetic demands of reproduction [43] and accumulate large fat reserves critical for survival during the long, cold winter [44]. Svalbard reindeer exhibit the largest seasonal amplitude in daily resting heart rate recorded in any ungulate [34] and downregulate metabolic rate during winter even when fed *ad libitum* in captivity [45]. Despite such strong adaptations to seasonal energetic constraints, there is considerable uncertainty in how flexible they are in their response to short-term environmental and physiological challenges.

We deployed internal biologging devices to measure heart rate and subcutaneous body temperature (*T*_sc_) in 19 adult female Svalbard reindeer of known body mass, age and reproductive status. In addition, animals were fitted with a global positioning system (GPS) collars containing activity sensors. Using these data, we quantified variation in heart rate in relation to animal age, reproductive status, body mass, *T*_sc_, activity level and environmental temperature within the seasonal peak (summer) and trough (winter) of heart rate. Identifying the correlates of intraseasonal variation in metabolic rate, as indexed by heart rate, is important for understanding the challenges faced by Svalbard reindeer in a rapidly warming Arctic [46].

## 2. Methods

### (a) Study area and animals

The study was conducted in Nordenskiöld Land, Svalbard (77°50′–78°20′ N, 15°00′–15°60′ E). At this latitude, there is continuous daylight from 19 April to 23 August and continuous darkness from 14 November to 29 January. The plant growing season typically lasts from June until late August [47]. Monthly mean air temperatures in July 2018 and January 2019 were 6.8 ± 1.5°C and –10.1 ± 5.5°C, respectively (Svalbard airport, SN99840; http://eklima.met.no). Further information on the study system is provided in the electronic supplementary material, §1.a. Gestation in Svalbard reindeer lasts for approximately 7.5 months from October until calving in early June [8]. Peak lactation is expected 3–5 weeks postpartum based on domestic reindeer *Rangifer t. tarandus* [48]. We selected July as the representative month for mid-summer due to the seasonal peak in heart rate, and January to represent mid-winter. During these periods, circadian rhythmicity is weak [34].

### (b) Animal capture and data collection

Adult females (ages 5–8 years, marked as calves) were captured in March–April 2018 for biologger deployment and in April 2019 for biologger retrieval. On both occasions, animals were caught by net using snowmobiles [49]: we recorded their body mass (±0.5 kg) and checked for pregnancy using an ultrasound scanner (Kaixin Electronic Instrument Co., Xuzhou, China). Body mass and age were not correlated (*r* = −0.07, *p* = 0.9). In August 2018, surveys were conducted on foot to relocate marked animals and assess calf status. Eight out of 19 individuals were not observed, and their calf status was inferred from pregnancy status in April and activity pattern in early June as described in [50] (electronic supplementary material, table S1). Omitting individuals with inferred calf status resulted in similar parameter estimates and *p*-values in the analyses described below. All females with a calf at heel in August were classified as lactating.

### (c) Biologger programming, deployment and retrieval

We fitted each animal with a combined heart rate and temperature logger (DST centi-HRT, Star-Oddi, Gardabaer, Iceland; approximately 19 g), which was implanted subcutaneously on the left side of the sternum or behind the left axilla, while animals were under anaesthesia. Surgical procedures are described in the electronic supplementary material, §1.b. Heart rate was automatically calculated from a 4 s electrocardiogram (ECG) at 150 Hz measurement frequency and stored alongside a quality index of signal clarity. We programmed the loggers to record heart rate and subcutaneous body temperature (*T*_sc_) every 15 min, and to store a raw ECG signal every 6 h for manual validation. Validation and filtering steps are described in the electronic supplementary material, §1.c. Briefly, values were filtered based on minimum and maximum values that could be validated (20 and 175 beats per minute (bpm), respectively) and the loggers' internal quality assessment (keeping only high quality—level 0). On average, 63% of recordings per animal per day were retained for analysis. *T*_sc_ was recorded with an accuracy of 0.2°C and calibrations were conducted by the manufacturer prior to implantations and validated again 12–13 months later, after removal. After retrieval, data were downloaded with the Mercury software program and a communication box [51]. Of the animals recaptured in April 2019, nine had uninterrupted recordings of heart rate and *T*_sc_ available for the whole year, while ten stopped recording before January due to battery failure. Consequently, summer analyses (July 2018) were based on the data available for all 19 females, while winter analyses (January 2019) relied on data for nine animals. Subsetting the summer data using just the nine individuals from the winter dataset resulted in qualitatively similar results.

The animals were also fitted with a collar (Vertex Plus, Vectronic Aerospace GmbH, Berlin, Germany, approximately 750 g) containing a GPS receiver, an activity sensor and an Iridium Communication (satellite) system. The GPS receiver had a fix rate of 8 h and was used to locate animals prior to capture. The activity sensor measured acceleration along two orthogonal axes representing back–forward and right–left movements at 4 Hz intervals. An internal algorithm calculates activity as the difference in acceleration between two consecutive measurements and is given within a relative range between 0 and 255, providing a mean value of acceleration in each axis every 5 min [52]. Because heart rate was recorded every 15 min, we used the sum of all activity recorded in both axes between two heart rate timestamps. For example, for a heart rate recorded at 16 : 15, we used the sum of activity recorded at 16 : 05, 16 : 10 and 16 : 15. Activity values therefore ranged between 0 and 1530, where 0 represented no activity and 1530 maximum activity. We categorized behaviour into resting/stationary (less than 50; hereafter ‘resting’) and moving (≥50; hereafter ‘active’) based on the bimodal distribution of activity data (details provided in the electronic supplementary material, §1.d).

In the main valley of our study area, we had a black bulb thermistor (15 cm in diameter) containing an iButton temperature logger (iButton Link) situated 1.5 m above the ground. These black spheres are designed to measure effective environmental temperatures, as the temperature inside the black bulb is influenced by solar radiation, wind chill and precipitation in addition to air temperature [53]. Temperatures were recorded every 4 h throughout the study period. Therefore, we matched recordings by 2 h in each direction of the time stamp to match with heart rate and *T*_sc_ records. For example, black bulb temperature recorded at 1600 h was matched to all heart rate and *T*_sc_ records between 1400 and 1800 h. Information about the construction of the black bulb is provided in electronic supplementary material, §1.e. Hereafter, black bulb temperatures are referred to as effective environmental temperature (*T*_e_).

### (d) Statistical analyses

All statistical analyses were conducted in R v. 4.0.0 [54]. First, we modelled heart rate over the whole year (*n* = 393 708 recordings in total) with a generalized additive mixed-effects model using the ‘*bam’* function for large datasets [55] to analyse the seasonal trend. We fitted heart rate as the response variable and time (days) as a thin plate regression spline with smoothing parameter *k* = 20 and a penalization value (*λ*) of 1.4 [55]. *k* was selected and assessed using the ‘*gam.check’* function from the ‘mgcv’ package. An individual term was fitted as both random slope and intercept. We used an autoregressive structure (AR1) to account for intraindividual temporal autocorrelation. Since the filtering of recordings left missing values in the dataset, we added a weighting parameter that gave missing values a weight of 0 and non-missing values a weight of 1.

Second, we investigated the drivers of variation in resting and active (defined above) heart rate during July and January with separate models for each activity level and season. We used a linear mixed-effects (lme) model using the ‘nlme’ package with the individual as a random intercept and fitted an AR1 structure as described above. All models were initially fitted with the same explanatory variables using maximum likelihood and simplified through a stepwise backward model selection approach [56], with a likelihood ratio test performed at each removal step (electronic supplementary material, tables S2–S5). The explanatory variables fitted were time (calendar day), *T*_e_, body mass recorded during capture (April 2018 for summer models, March 2019 for winter models), reproductive status (lactation status in summer, pregnancy in winter; categorical ‘yes/no’) and age, *T*_sc_, as well as several biologically relevant interactions between the variables. For the models of active heart rate, an additional term for activity (continuous values from 50 to 1530) was fitted, together with additional interactions between activity and other variables (all parameters are listed in the electronic supplementary material, tables S2–S5). The final models were fitted with restricted maximum likelihood to account for random effects [56]. All continuous variables (activity, body mass, *T*_e_, *T*_sc_ and time) except age were scaled within seasons to a mean of zero and standard deviation relative to the variance. Time was fitted as a quadratic term in the summer models to account for the peak in heart rate in mid-July and as a linear term in the winter models. We used the function ‘*rsquared.GLMM’* from the ‘MuMIn’ package to derive the coefficients of determination (*R*^2^) for fixed effects (marginal *R*^2^) and fixed and random effects combined (conditional *R*^2^) to assess the amount of variation explained by each model [57]. The generation of figures from model predictions is described in the electronic supplementary material, §2.c.

## 3. Results

**Figure 1.**
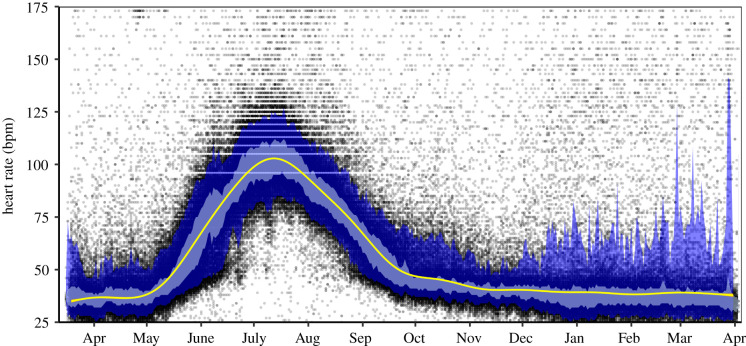
Annual variation in heart rate across activity and reproductive states in Svalbard reindeer females, based on the data for 19 individuals (sample size declines throughout the year; see §2 for details). The *x*-axis spans from March 2018 to April 2019, and each tick mark indicates the first day of the month. The solid yellow line represents predictions of heart rate across all individuals, fitted with a generalized additive mixed model as a function of time with the individual as a random term (*R*^2^ = 0.75). The black points are raw data values; the dark blue area represents values within the lower 5% and upper 95% quantiles, and the light blue area represents values within the lower 25% and upper 75% quantiles of the data.

Predicted heart rates from the generalized additive mixed-effects model peaked in mid-July at 103 bpm, declined to 40 bpm in December and then remained relatively stable until April, when the loggers were removed (figure 1). Day of the year and individual variation explained 75% of the variation in heart rate. In both winter (January) and summer (July), arithmetic means of heart rate were 10 bpm lower when resting compared to active heart rate (electronic supplementary material, table S2, and figure S9). In winter, animals were active 44% of the time compared to 66% in summer (electronic supplementary material, table S2).

The lme models of winter heart rate explained relatively little variation. Together, fixed and random effects explained only 5% (fixed effects 2%) and 7% (fixed effects 6%) for resting and active heart rate, respectively. The summer lme models explained considerably more variation in heart rate, accounting for a total of 42% (fixed effects 16%) of variation in the resting state and 38% (fixed effects 24%) of variation in the active state. Despite the marked difference in the fit of the final models in each season, there were many similarities in the model parameters included. Therefore, we continue by describing the explanatory variables from lme models in turn, presenting results of model predictions with 95% confidence intervals (CI) in parentheses.

**Figure 2.**
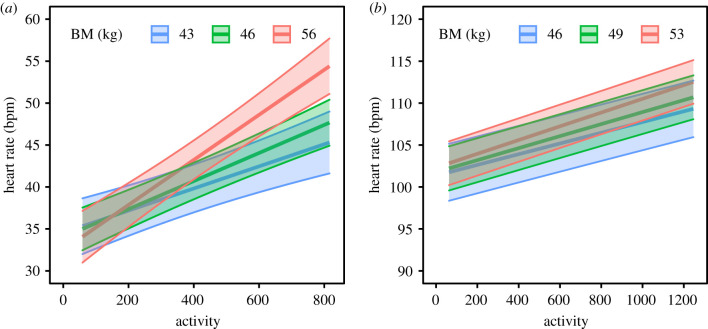
Predicted heart rate (±95% CI) of Svalbard reindeer females*,* plotted against activity levels in interaction with body mass grouped based on the 0.15, 0.5 and 0.85 quantiles of the distribution of body mass in (*a*) winter and (*b*) summer.

Body mass did not influence heart rate in resting animals in either summer or winter (table 1). In the active state, however, the increase in heart rate with increasing activity was greater in heavier animals (table 1). In winter, predicted heart rate rose from 34 bpm (CI: 31–39) to 54 bpm (CI: 51–58) at the highest activity levels in heavy (approx. 56 kg) individuals compared with a rise from 35 bpm (CI: 32–39) to 45 bpm (CI: 42–49) in light individuals (approx. 43 kg, figure 2*a*). This interaction was also significant in summer (table 1), but less pronounced with only a 4 bpm difference between heavy individuals at 113 bpm (CI: 110–115) and light individuals at 109 bpm (CI: 106–113) at the highest activity levels (figure 2*b*). In the active state, body mass also interacted with *T*_sc_, but only in winter (table 1). Whereas lighter animals reduced heart rate markedly from 47 bpm (CI: 44–50) at *T*_sc_ 37°C to 28 bpm (CI: 21–34) at *T*_sc_ 31°C, heavy individuals maintained a constant heart rate at approximately 44 bpm (CI: 41–47) across the range of *T*_sc_ (electronic supplementary material, figure S10).

**Table 1.** Results of linear mixed-effects models on the heart rate of Svalbard reindeer females, during rest and while active in both summer (July) and winter (January). The values are parameter estimates of the fixed effects, with upper and lower 95% CIs given in brackets. The standard deviations of the random intercepts of each model were 1.2 and 2.1 for resting and active in winter, and 5.6 and 4.4 for resting and active in summer, respectively. All continuous variables except age were scaled with a mean of 0 within each season. The final models were fitted with restricted maximum likelihood. Sample size ‘*N*’ represents the number of unique individuals, while ‘*n*’ represents the number of unique observations. For each model, reference levels for the intercepts are based on non-reproductive females (0). BM, body mass (kg); ‘–‘, not applicable/tested in model; ns, not significant (removal based on maximum likelihood ratio test); RS, reproductive status (1 = lactating in summer or pregnant in winter, 0 = non-reproductive); *T*_e_, environmental temperature; *T*_sc_, subcutaneous body temperature.

model parameters (fixed effects)	summer (*N* = 19)		winter (*N* = 9)	
	resting (*n* = 11 287)	active (*n* = 24 436)	resting (*n* = 8936)	active (*n* = 4495)
intercept	113.8 (98.2, 129.5)	126.3 (113.9, 138.6)	33.2 (28.0, 38.5)	48.7 (37.8, 59.6)
time (days)	−0.5 (−0.7, −0.3)	−1.9 (−2.1, −1.7)	0.4 (0.2, 0.5)	0.2 (−0.3, 0.9)
time (days)^2^	−1.9 (−2.1, −1.7)	−2.1 (−2.3, −2.0)	–	–
activity	–	3.2 (3.0, 3.4)	–	5.2 (4.3, 6.1)
age	−3.4 (−6.0, −0.8)	−3.9 (−6.0, −1.9)	−0.1 (−1.2, 0.9)	−0.8 (−2.9, 1.3)
BM	ns	1.4 (−1.1, 3.9)	ns	0.4 (−1.7, 2.6)
RS (1)	3.8 (−2.1, 9.7)	3.6 (−1.1, 8.3)	2.0 (−0.3, 4.4)	−5.9 (−12.8, 1.1)
*T*_e_	−0.7 (−0.9, −0.6)	−0.2 (−0.4, −0.1)	−0.2 (−0.3, −0.1)	−0.8 (−1.3, −0.2)
*T*_sc_	−2.3 (−3.2, −1.4)	−3.3 (−4.4, −2.4)	−0.8 (−1.7, 0.1)	1.1 (−7.8, 10.0)
activity × BM	–	0.5 (0.3, 0.8)	–	1.4 (0.5, 2.3)
activity × *T*_sc_	–	0.3 (0.1, 0.5)	–	3.8 (2.4, 5.3)
age × *T*_sc_	0.4 (0.2, 0.5)	0.6 (0.4, 0.7)	0.2 (0.0, 0.4)	−1.5 (−2.7, −0.3)
BM × *T*_sc_	ns	ns	ns	−2.5 (−3.8, −1.3)
RS (1) × *T*_sc_	0.6 (0.3, 1.0)	1.4 (1.0, 1.8)	ns	8.2 (2.2, 14.1)

**Figure 3.**
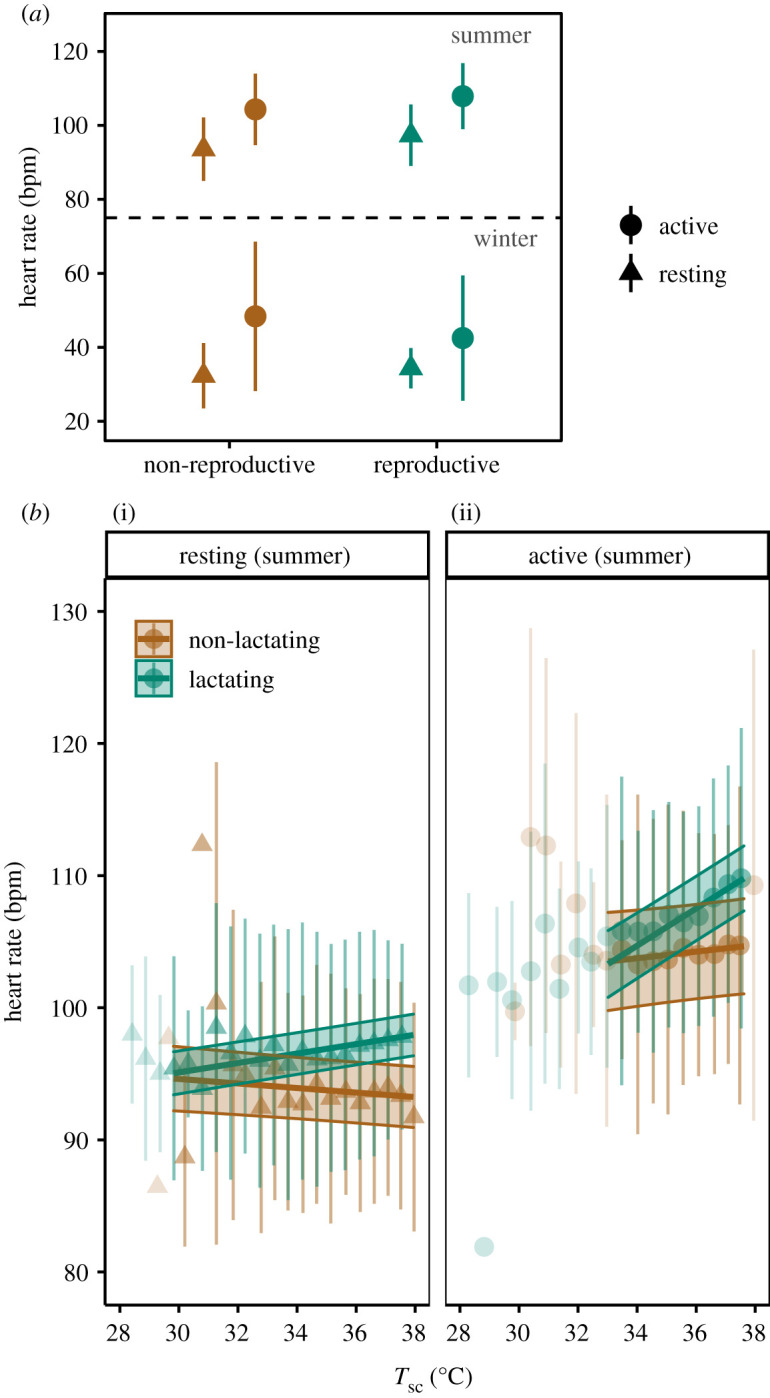
Predicted heart rate for reproductive (green) and non-reproductive (brown) females while resting (triangles) and while active (circles). (*a*) Predicted heart rate (±s.e.) grouped by reproductive state (summer: lactating *N* = 13, non-lactating *N* = 6; winter: pregnant *N* = 7, non-pregnant *N* = 2), activity state and season. (*b*) Predicted heart rates (±95% CI) for lactating (green) and non-lactating (brown) Svalbard reindeer females in summer, while resting (i) and while active (ii) in response to subcutaneous body temperature (*T*_sc_,°C). Points and their error bars represent mean ± s.d. of heart rate adjusted for the other model predictors (table 1). Points that fall outside the predicted range are values below the lower 0.01 and above the upper 0.99 quantiles of the *T*_sc_ distribution.

Reproductive status affected heart rate in summer and during activity in winter. The effect size in summer, when lactating, was greater (on average 4 bpm higher in reproductive females) than in winter when pregnant (2 bpm difference) (figure 3*a*). The reproductive status also interacted with *T*_sc_, but differently for summer and winter (table 1). During summer, lactating females had higher heart rates at higher *T*_sc_, both during activity and at rest (table 1, figure 3*b*). In winter, the interaction between reproductive status and *T*_sc_ was only significant when active (table 1), with the heart rate of pregnant females displaying both a positive relationship with, and a greater range of *T*_sc_ (electronic supplementary material, figure S11). Finally, in summer, lactating females spent on average 6% more time active (67% versus 61% in non-lactating females, *p* < 0.001), whereas in winter, there was no significant difference in time spent active between the two reproductive groups (electronic supplementary material, table S2).

**Figure 4.**
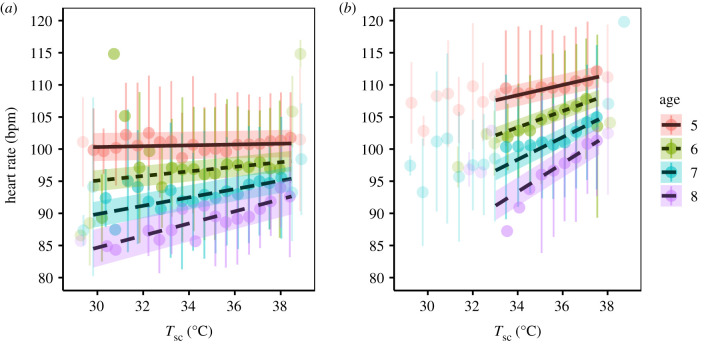
Predicted heart rate (±95% CI) plotted against subcutaneous body temperature (*T*_sc_,°C) in interaction with age (5-, 6-, 7- and 8-year olds) in summer (*a*) during rest and (*b*) while active. Points and their error bars represent mean ± s.d. adjusted the other model predictors (table 1). Points that fall outside the predicted range are values below the lower 0.01 and above the upper 0.99 quantiles of the *T*_sc_ distribution.

Age had a pronounced effect on heart rate in summer, regardless of reproductive and activity states. Predicted heart rate declined by 10 (resting) and 12 (active) bpm in 8-year olds compared to 5-year olds (table 1). Furthermore, there was an interaction between age and *T*_sc_ during summer, with a greater effect in older animals. When resting, an 8-year old who lowered *T*_sc_ to 30°C had a predicted heart rate of 84 bpm (CI: 82–87) compared to 100 bpm (CI: 98–102) in a 5-year old (figure 4). When active, the magnitude of the age difference was again greater at lower *T*_sc_ (figure 4). The interaction between age and *T*_sc_ was also significant in winter; however, differences were small (differences of 1–2 bpm) and no 8-year olds were present in the winter dataset (electronic supplementary material, table S1 and figure S12).

Being active raised heart rate as expected, but relatively more so in winter than in summer (table 1). However, the effect of activity on heart rate interacted with body mass, as described above (figure 2), and furthermore with *T*_sc_, especially in winter (table 1). The relation of heart rate with *T*_sc_ was greatest at high activity levels and virtually absent at low activity levels (electronic supplementary material, figure S13a). Conversely, in summer, the relation of heart rate with *T*_sc_ was more similar at various activity levels, albeit still significantly different (table 1; electronic supplementary material, figure S13b).

Declining environmental temperature (*T*_e_) raised heart rate in both winter and summer. The effect was slightly more pronounced when active compared to resting in winter, with predicted differences of 2.4 bpm and 0.7 bpm, respectively, when *T*_e_ declined from −2°C to −22°C (electronic supplementary material, figure S14). In summer, the effect was greater when resting compared to active: predicted difference of 4.2 bpm and 1.2 bpm, respectively, when *T*_e_ declined from 25°C to 4°C (electronic supplementary material, figure S14).

## 4. Discussion

Our results demonstrate that the impact of physiological and environmental factors on heart rate depends on both individual state and seasonal context. In particular, the relationship between heart rate and subcutaneous body temperature (*T*_sc_) suggests that peripheral heterothermy, i.e. variability in the body ‘shell’ temperature [58], is an important mechanism for energy management, not only in winter, but also in summer. Furthermore, several interactions indicate that the benefit of heterothermy is dependent on activity, body mass, reproductive status and age, especially in summer. The most common drivers of energy expenditure such as reproduction [59] and activity [60] had only small effects on heart rate within the seasons (2–6% in winter and 16–24% in summer), whereas seasonality itself explained a large amount of the variation (75%) in annual heart rate. This seasonality is consistent with a strong selective pressure on energy conservation in winter and maximizing energy intake in summer for reproduction and replenishment of body reserves. Here, we discuss the observed energy management mechanisms that contribute to meeting these seasonal energetic constraints.

Endogenous heat production is an inevitable energetic cost for endotherms, and mechanisms to reduce this cost can be of great importance, particularly during periods of energy deficits and low ambient temperatures [15]. Reductions in heart rate have been strongly associated with a decrease in both core [25,34] and peripheral body temperatures [40,61,62]. We found that heart rate and *T*_sc_ were correlated in both seasons. Interestingly, this association was greater (more positive) in individuals with lower body mass in winter and in older females in summer. Peripheral heterothermy is likely to be an important mechanism to reduce heat loss and save energy by minimizing the temperature gradient between the body shell and the environment [58,63]. Winter body mass in Svalbard reindeer is strongly influenced by the size of the fat stores [8]. Body condition (indexed by fat stores) begins to decline around age seven *(Pigeon, unpublished data)*, at an age when teeth also start to wear down, which may cause a lower rate of energy assimilation [64] due to larger particles and longer retention time in the digestive system [65]. Our results indicate that peripheral heterothermy may be more pronounced in individuals of poorer body condition in winter, or with limitations on food intake in summer. Indeed, state-dependent heterothermy has been demonstrated in moose, where individuals of poor body condition displayed lower core body temperature [66]. Furthermore, the degree of hypothermia in both peripheral [67,68] and core body temperature [69] has been shown to increase in response to food restriction. Plasticity in the ability to employ heterothermy may therefore be a key factor that enables animals to respond to short-term energy deficits or limitations in energy uptake [12].

Reproduction, and lactation in particular, is considered the most energetically demanding part of the annual cycle for female mammals [70] and has been proposed as the main driver of seasonally elevated mammalian energy expenditure [71]. Although we found a significantly higher heart rate in lactating compared to non-lactating female Svalbard reindeer, the difference was surprisingly small (approx. 6%) and heart rates of non-lactating females were still more than twice that of winter rates (figure 2*a*). Our results demonstrate that elevated heart rate in summer is largely independent of reproduction, as has been observed in other seasonal animals [25,41]. The most likely explanation for the seasonal increase is the relatively narrow time window when forage is abundant, requiring a substantial upregulation of the metabolic machinery in order to recover body reserves and ensure survival during the coming winter, regardless of reproductive state [34]. The energetic cost of lactation is mainly determined by the amount of energy exported through the milk and is not necessarily reflected in an elevation of the total metabolic rate [72]. Further, the simultaneous increase of heart rate with *T*_sc_ found in lactating females only (figure 2*b*) may indicate that lactating females are constrained in dissipating surplus heat and thus are more susceptible to heat stress in summer [17]. Altogether, the relatively small increase of heart rate associated with lactation suggests that females may compensate for the additional cost of lactation by downregulating other metabolic processes such as ‘background’ metabolic rate [72,73] or replenishment of fat reserves [74]. In addition, lactating females were more active than non-lactating females in summer, suggesting higher foraging activity in response to elevated energy demands. However, this behavioural response is apparently not able to compensate fully for lactational costs as females that do not raise a calf have been found to be heavier, i.e. fatter, in autumn than those that reproduced successfully [8,74].

Although an increase in heart rate with increasing activity levels occurred in both seasons, in line with previous studies in other *Rangifer* subspecies [75], the relative increase in mean heart rate from resting to active was much greater in winter than in summer (29% versus 10%). Walking through snow and cratering in snow are both energetically costly activities and likely contribute to relatively greater increases in heart rate during activity in winter compared to summer [76,77]. This effect was even greater in heavier females, suggesting that the cost of locomotion increases disproportionally with body mass and the intensity of activity [78]. Also, the reduced time spent active in winter is indicative of behavioural compensation to reduce energy deficits during periods of low food supply [79]. In summer, a higher proportion of time spent foraging [42] is likely to lead to a greater degree of rumen filling and, in turn, precipitate increased energy uptake and necessary increase in blood supply to the rumen [80] contributing to increased heart rates, even when resting.

Overall, the strong seasonal pattern in heart rate contributes to the increasing evidence that seasonal animals upregulate energy expenditure in periods of high supply and downregulate it when food is scarce [22–24]. The relatively small elevations in heart rate in lactating females could indicate that breeding female reindeer are close to their upper limits of sustained metabolic rate in summer. Furthermore, the low proportion of variance explained in winter heart rate may indicate that Svalbard reindeer operate close to their lower limits of metabolic rate, a limit that may also be dictated by the cost of maintaining high core body temperature to maintain the rumen biota [80], and that is reflected in the high mortality observed in winters with severely restricted food access [81]. While enhanced insulation in winter counteracts thermoregulatory challenges in endotherms exposed to low ambient temperatures [13], a negative relationship between ambient temperature and heart rate within seasons suggests that thermoregulatory responses to low temperature are still present even in highly seasonal animals, albeit at a much smaller scale compared to the seasonal effect [5,39,41]. This could indicate that intraseasonal and interseasonal responses to environmental variation can differ within a species [42]. While the seasonal plasticity in energetics makes Svalbard reindeer well-adapted to their highly seasonal environment, intraseasonal constraints on yet further upregulation or downregulation of heart rate may limit their ability to respond to severe environmental change [12].

## 5. Conclusion

Here, we have highlighted the intraseasonal responses in heart rate to short-term environmental and physiological changes in a high-Arctic ungulate. We find that energy-saving mechanisms such as peripheral heterothermy depend on body condition, age and reproductive state. Overall, a strong seasonal pattern overshadowed relatively small intraseasonal responses in heart rate, emphasizing the importance of evaluating individual state and seasonal context when studying energetics in free-living animals [79].

## Data accessibility

Data are available from the Dryad Digital Repository: https://doi.org/10.5061/dryad.mcvdnck0f [82].
